# Patient-specific mental rehearsal with interactive visual aids: a path worth exploring?

**DOI:** 10.1007/s00464-017-5788-2

**Published:** 2017-08-24

**Authors:** Marina Yiasemidou, Raffaele Galli, Daniel Glassman, Matthew Tang, Rahoz Aziz, David Jayne, Danilo Miskovic

**Affiliations:** 1 Leeds Institute of Biomedical and Clinical Sciences, University of Leeds, Leeds, UK; 2 John Goligher Surgery Unit, St. James University Hospital, Leeds, UK; 3 Bradford Teaching Hospitals, Bradford, UK; 4 Medical School, University of Leeds, Leeds, UK

**Keywords:** Mental rehearsal, Pre-operative preparation, Patient-specific, Surgical skills

## Abstract

**Background:**

Surgeons of today are faced with unprecedented challenges; necessitating a novel approach to pre-operative preparation which takes into account the specific tests each case poses. In this study, we examine patient-specific mental rehearsal for pre-surgical practice and assess whether this method has an additional effect when compared to generic mental rehearsal.

**Methods:**

Sixteen medical students were trained how to perform a simulated laparoscopic cholecystectomy (SLC). After baseline assessments, they were randomised to two equal groups and asked to complete three SLCs involving different anatomical variants. Prior to each procedure, Group A practiced mental rehearsal with the use of a pre-prepared checklist and Group B mental rehearsal with the checklist combined with virtual models matching the anatomical variations of the SLCs. The performance of the two groups was compared using simulator provided metrics and competency assessment tool (CAT) scoring by two blinded assessors.

**Results:**

The participants performed equally well when presented with a “straight-forward” anatomy [Group A vs. Group B—time sec: 445.5 vs. 496 *p* = 0.64—NOM: 437 vs. 413 *p* = 0.88—PL cm: 1317 vs. 1059 *p* = 0.32—per: 0.5 vs. 0 *p* = 0.22—NCB: 0 vs. 0 *p* = 0.71—DVS: 0 vs. 0 *p* = 0.2]; however, Group B performed significantly better [Group A vs. B Total CAT score—Short Cystic Duct (SCD): 20.5 vs. 26.31 *p* = 0.02 *η* ^2^ = 0.32—Double cystic Artery (DA): 24.75 vs. 30.5 *p* = 0.03 *η* ^2^ = 0.28] and committed less errors (Damage to Vital Structures—DVS, SCD: 4 vs. 0 *p* = 0.03 *η* ^2^=0.34, DA: 0 vs. 1 *p* = 0.02 *η* ^2^ = 0.22). in the cases with more challenging anatomies.

**Conclusion:**

These results suggest that patient-specific preparation with the combination of anatomical models and mental rehearsal may increase operative quality of complex procedures.

Driven by patient safety issues most western countries imposed working hours’ restrictions [1, 2] in order to reduce medical errors made by fatigued doctors working long hours [3]. Since their introduction, avoidable medical errors and adverse events have decreased [4, 5] and surgical residences’ quality of life improved [6, 7]. However, alongside working hours, training time and opportunities were condensed [3]. As a result, conventional training patterns, purely based on exposure to a rich and diverse clinical case mix has become unrealistic.

Combined with increasing technological advancements dominating contemporary surgery [8], training requirements have radically changed in the past decades [9]. Although “in-vitro” methods such as simulation were shown to successfully increase technical skills [8] methods that can increase the efficiency of available training time in a clinical environment have not been adequately explored. Techniques such as mental practice [10] or patient-specific surgical rehearsals, have been trialled for increasing efficiency and quality of surgery [11–15] but to this date have not gained widespread recognition.

Cognitive reproduction of a motor task without explicit physical movement, otherwise known as mental rehearsal or imagery [16–18], has been successfully used in various fields [19–21], including surgery [22–27], for the acquisition of motor skills. The similarity of neurocognitive pathways activated during mental and real practice of a motor task is increasingly being recognised by electroencephalography studies [28–30]. The content of mental rehearsal sessions in surgery is variable. Most commonly, it takes the form of relaxation techniques followed by a step-by-step breakdown of the procedure, or a descriptive text, inclusive of visual and kinaesthetic cues [23, 24, 27, 31, 32] derived from semi-structured interviews with expert surgeons [23, 31]. This process is performed once [24, 27, 32] or repeated several times [25, 33, 34] in order to prepare for the actual surgical procedure.

Mental rehearsal does not usually involve operation-specific characteristics, which are important, as they often determine the technical difficulty of an operation. Some operation specifics (e.g. anatomical variations) can be derived from medical imagery pre-operatively and incorporated into the surgeon’s preparation, facilitating a more precise representation of intraoperative difficulties. Introduction of patient-specific elements into mental rehearsal can be readily achieved with the use of patient-specific anatomical models.

Surgical planning using patient-specific anatomical models has been sporadically applied in the past [11, 13, 15, 35–61] and although it is more popular in some specialties [11, 12, 35, 36, 39–41, 43, 46, 50–52, 62–65], it has not penetrated into routine practice. Some of the reasons for this are the cost of associated hardware and the time required in the simulation suite [11, 12, 15]. The fusion of mental rehearsal and anatomical models does not require the use of a simulator and can be practiced repeatedly in the surgeon’s own time using a personal computer.

The authors have previously assessed the feasibility of combining mental rehearsal and patient-specific interactive anatomical models [66], but have not explored this modality within technically demanding cases. The current study aims to evaluate whether the addition of interactive case/patient-specific element to mental rehearsal can provide an additional benefit to mental rehearsal alone.

## Methods

### Surgical procedure

For the purposes of this exploratory study, simulated laparoscopic cholecystectomy (LC) was the procedure of choice for the following reasons: (i) virtual reality LC simulators are readily available (LapMentor^®^, Simbionix, Israel) [67], (ii) simulated operations with anatomical variations are provided [68], (iii) LC is a commonly performed operation involving complex laparoscopic skills [69], and (iv) the anatomy of the cystic duct and artery vary significantly, demanding varying degrees of technical competency [70].

### Participants

Sixteen medical students, (years two–five and intercalating), who have never seen a laparoscopic cholecystectomy or used the virtual reality simulator before, volunteered for the study after receiving email invitation using the mailing list of the university of Leeds. Sample size calculation was based on the primary outcome for the study, the Competency Assessment Tool—CAT, a validated scoring system for assessing surgical performance, specifically designed for laparoscopic cholecystectomy [71]. A reduction in CAT score from 3 to 2 was assumed to be clinically meaningful, requiring 8 patients to be recruited to either Group A using a mental rehearsal checklist to prepare prior to simulated surgery or Group B using the same checklist and an interactive 3D anatomical model; to determine a significant difference at 80% power (α=0.05, β=0.2, Standard Deviation of 0.7).

Subjects underwent small group teaching sessions on the clinical indications, anatomy, surgical technique, and complications after a laparoscopic cholecystectomy (LC). They were shown how to use the virtual reality simulator (VRS) and taught a series of defined tasks on the simulator as well as a complete laparoscopic cholecystectomy. Subsequently, they performed 10 repetitions of the “normal anatomy” laparoscopic cholecystectomy, each at least 45 min apart from the other.

**Fig. 1:**
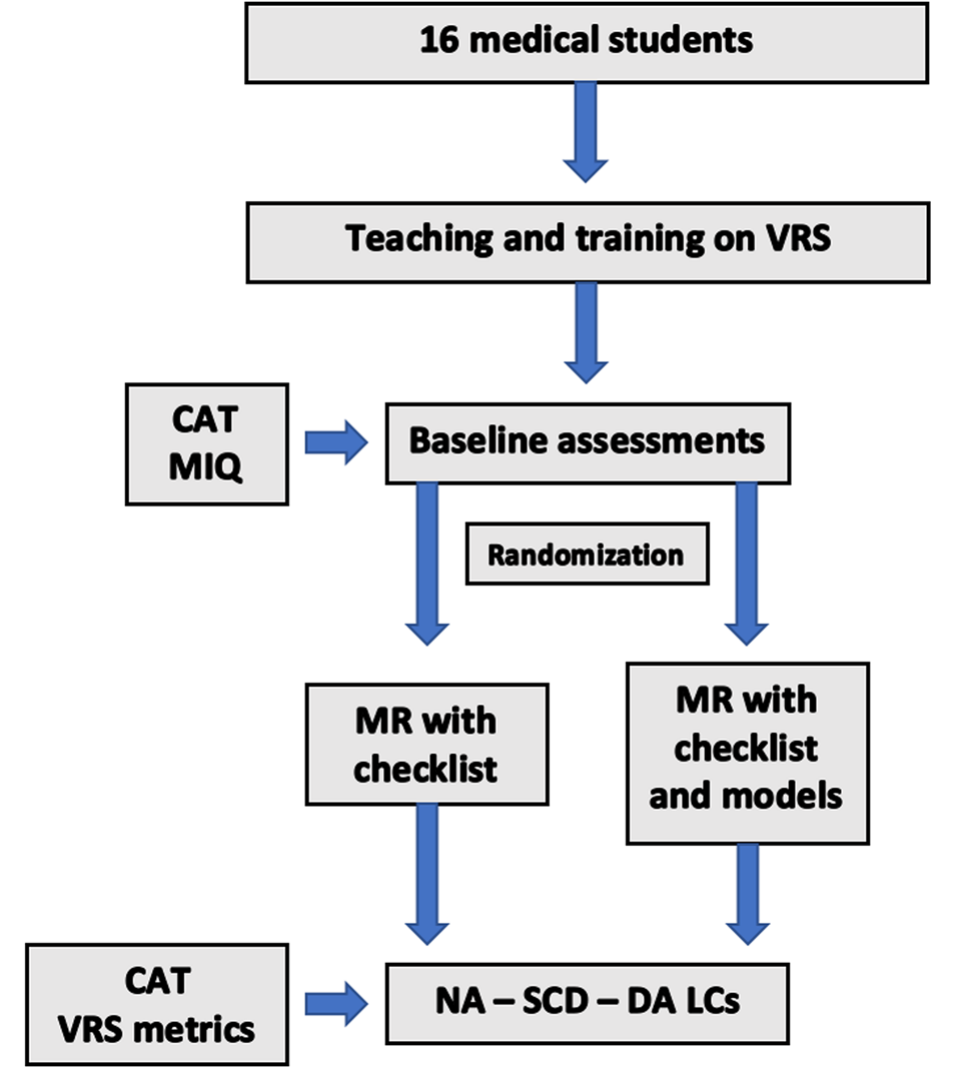
Study methodology. *VRS* virtual reality simulator, *CAT* competency assessment tool, *MR* mental rehearsal, *MIQ* mental imagery questionnaire, *NA* normal anatomy, *SCD* short cystic duct, *DA* double cystic artery

Upon conclusion of the training phase, participants completed a questionnaire assessing their ability for mental imagery (MIQ-RS) [72] and performed a simulated laparoscopic cholecystectomy, which was scored using CAT. The MIQ-RS consists of 14 tasks; trainees are initially asked to physically perform an action (e.g. raising a knee as high as possible and then lowering the knee so they are standing again on two feet) and after they are asked to visualise or to feel themselves performing the same task without overt physical movement. Subsequently, they were asked to score how easy it was to visualise or feel the task. A Likert scale (1–7, 1: Very hard to see/feel, 7: Very easy to see/feel) was used for that purpose [72]. According to the results of the MIQ-RS and CAT, they were paired in dyads of similar ability and then randomised to two equal groups (Fig. 1) through the process of a draw consisting of eight “checklist only” tickets and eight “checklist and model” tickets. Had participants within a couple drawn the same type of ticket, the process was repeated until they were randomised into two different groups. In such a manner the number of participants in each group remained equal.

## Preparation of mental rehearsal checklist

For the purposes of preparing a mental rehearsal checklist (Table 1) semi-structured interviews were conducted with five specialist surgeons who regularly perform laparoscopic cholecystectomy. The concepts of mental rehearsal, and visual and kinaesthetic cues were explained and they were asked to describe how they would perform a laparoscopic cholecystectomy.

**Table 1:** Mental rehearsal checklist

Step	Instruction	View model
1	Visualise the retracted liver and gallbladder	*
2	Decide which instruments to use and insert them into the “abdomen” under direct vision (visualise and feel)	
3	Visualise Calot’s triangle	*
4	Retract the gallbladder (feel) in a manner that highlights Calot’s triangle (visualise the retracted gallbladder)	*
5	Decide from where and how you will commence dissection	*
6	Begin dissecting Calot’s triangle (visualise and feel)	
7	Continue the dissection carefully exposing the cystic duct and artery while adjusting the place of the retracted gb to achieve optimal view—describe the movements of both hands (visualise and feel) and what are the end points of the dissection	*
8	Visualise the skeletonised artery and duct	*
9	Insert the clip applier under direct vision (visualise). Place firmly on the cystic duct (feel), visualise both jaws of the instrument (visualise) and then place the number of clips you wish, where you choose (visualise)	*
10	Repeat step 9 with artery—visualise the end result to ensure no complications occurred	*
11	Insert the electrocautery instrument you will use for dissecting the gall bladder off the liver bed under direct vision (visualise)	
12	Retract the gallbladder as you see fit (visualise and feel) and commence the dissection of the gb off the liver bed from the point you choose (visualise)	*
13	Continue the dissection of the gallbladder from the liver bed adjusting the retraction position as you see fit (visualise and feel)—describe your movements	*
14	Ensure that there is no bleeding from the liver bed either right before the completion of the dissection or at the end of it (visualise)—describe how you would deal with any bleeding	

**Fig. 2:**
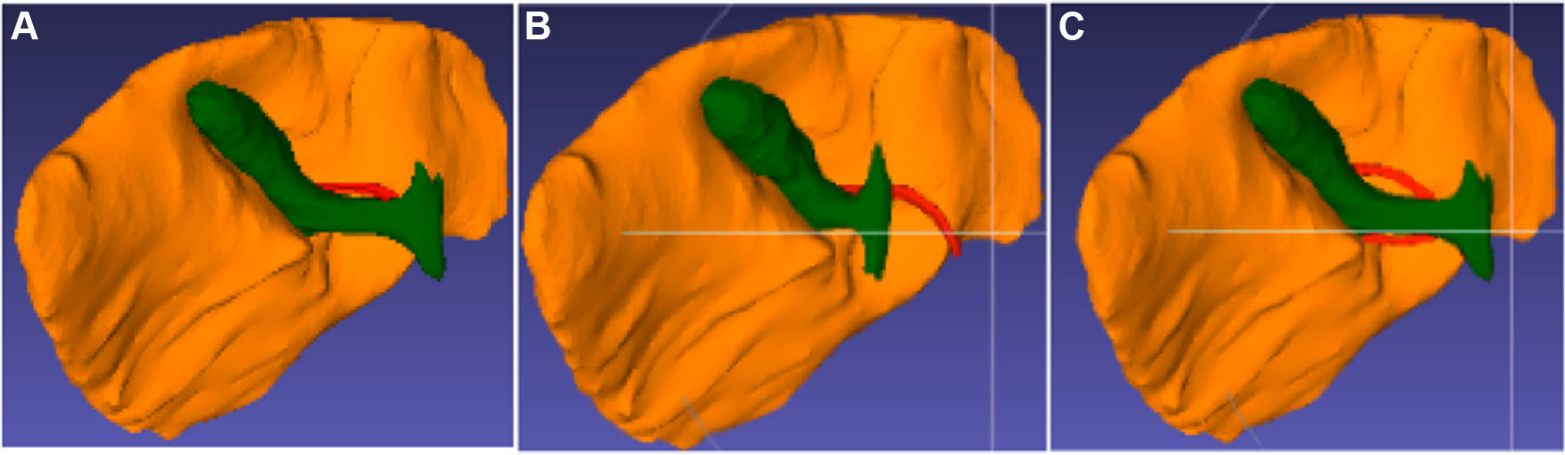
Virtual models **A** normal anatomy, **B** short cystic duct and **C** double cystic artery

The interviews were transcribed verbatim and analysed by two of the authors, conducting descriptive synthesis and extraction of visual (e.g. “I now see Calot’s triangle”) and kinaesthetic cues (e.g. “I retract the gallbladder towards the right shoulder with moderate strength”) embedded within various steps of the procedure. The most commonly occurring cues were introduced into the checklist. These were combined with the stages of the procedure most frequently described by the surgeons in order to produce a 14-step checklist (Table 1) which could be combined with visualisation of the interactive 3D models (Fig. 2). This was adjusted to the stages of the procedure which can be completed on the VRS.

### 3D models preparation

Three different anatomical variations were chosen for this study: “normal anatomy” (NA), “short cystic duct” (SCD) and “double cystic artery” (DA). For each anatomical variation, a 3D model was reconstructed manually from an anonymised computed tomography (CT) scan using an “in-house” 3D reconstruction software (Volume Viewer, University of Leeds). The model was exported onto open source visualisation software (MeshLab).

The NA gallbladder consisted of a normal sized cystic duct and a single cystic artery positioned posteriorly to the cystic duct. The SCD had a shorter duct and a single artery posterior to the duct. The DA gallbladder had a normal sized duct and two cystic arteries, one anterior and one posterior to the cystic duct (Fig. 2).

### Intervention and comparators

During the mental rehearsal session, the subjects were seated in a quiet place and given time to relax. Participants randomised to group B were taught how to use the 3D model viewing software. All subjects were asked to read through the mental rehearsal checklist and prepare to verbalise how they would perform the procedure whilst “viewing” and “feeling” the operation (visual and kinaesthetic cues) based on their previous experience of performing the procedure on the simulator.

The participants randomised to group A (*n* = 8) were asked to perform a Normal Anatomy (NA) simulated LC, a Short Cystic Duct (SCD) and a Double cystic Artery (DA) simulated LC after completing a mental rehearsal session with the use of the checklist only. The students randomised to group B (*n* = 8) were asked to do the same, but for most steps on the checklist (indicated with an asterisk—Table 1) they were also asked to review the appropriate anatomical model. Group A was informed of the anatomical variation of the eminent procedure, but did not have access to the relevant anatomical model provided to group B. This process was repeated before every simulated procedure. All procedures were video-recorded for later assessment.

### Measured outcomes

Performance (time, Number Of Movements—NOM and Path Length—PL) and safety metrics (Number of perforations—Per, number of Non-Cauterised Bleeding—NCB and number of Damages to Vital Structures—DVS) automatically provided by the VRS were compared between the two groups for each type of anatomy. Proficiency gain curves for time to complete the procedure (time), Number Of Movements [73] and Path Length (PL) of the instrumental tip were generated by curve fitting raw data using power law [f(x) = ax^k^ − a: first attempt result and k: log of learning rate divided by log of 2] [74].

The recordings of the procedure were judged by two blinded assessors [R.G, D.G] using the competency assessment tool designed specifically for laparoscopic cholecystectomy [71]. The initial category of this score refers to the insertion of ports and as this was not part of the VRS, this category was not used for scoring.

### Statistical analysis

The unpaired *t*-test was used to compare continuous data and the Mann–Whitney *U*-test for discrete data. Eta squared is reported for the statistically significant outcomes (*p* < 0.05). IBM^®^ SPSS^®^ Statistics Vs. 24 and GraphPad Prism^®^ 7.0b, GraphPad Software, Inc. were used for all statistical analysis and preparation of graphs. Agreement between assessors was evaluated using the Intraclass Correlation Coefficient (ICC).

## Results

**Fig. 3:**
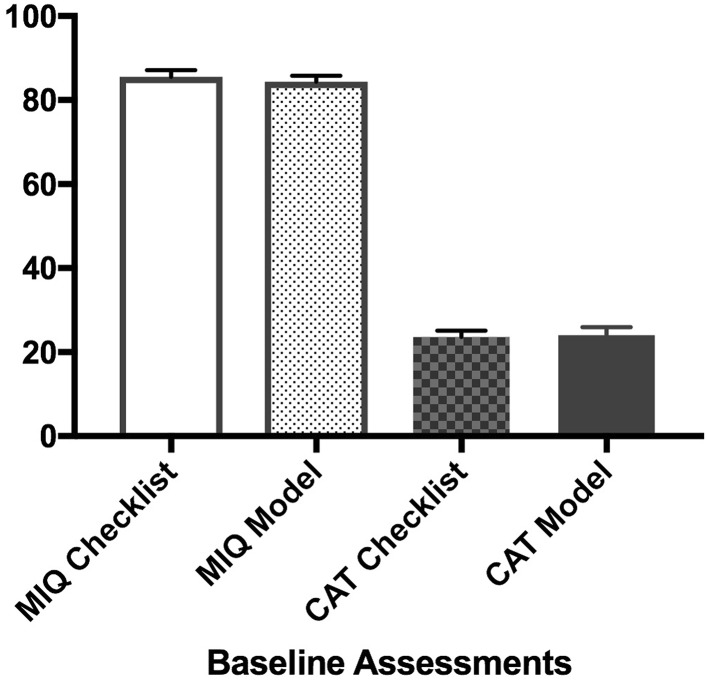
Baseline ability of the two groups. *MIQ* mental imagery questionnaire, *CAT* competency assessment tool. *Y*-axis demonstrates mean values for each variable indicated in the *X*-axis and *error bars* show SEM (standard error of mean)

**Fig. 4:**
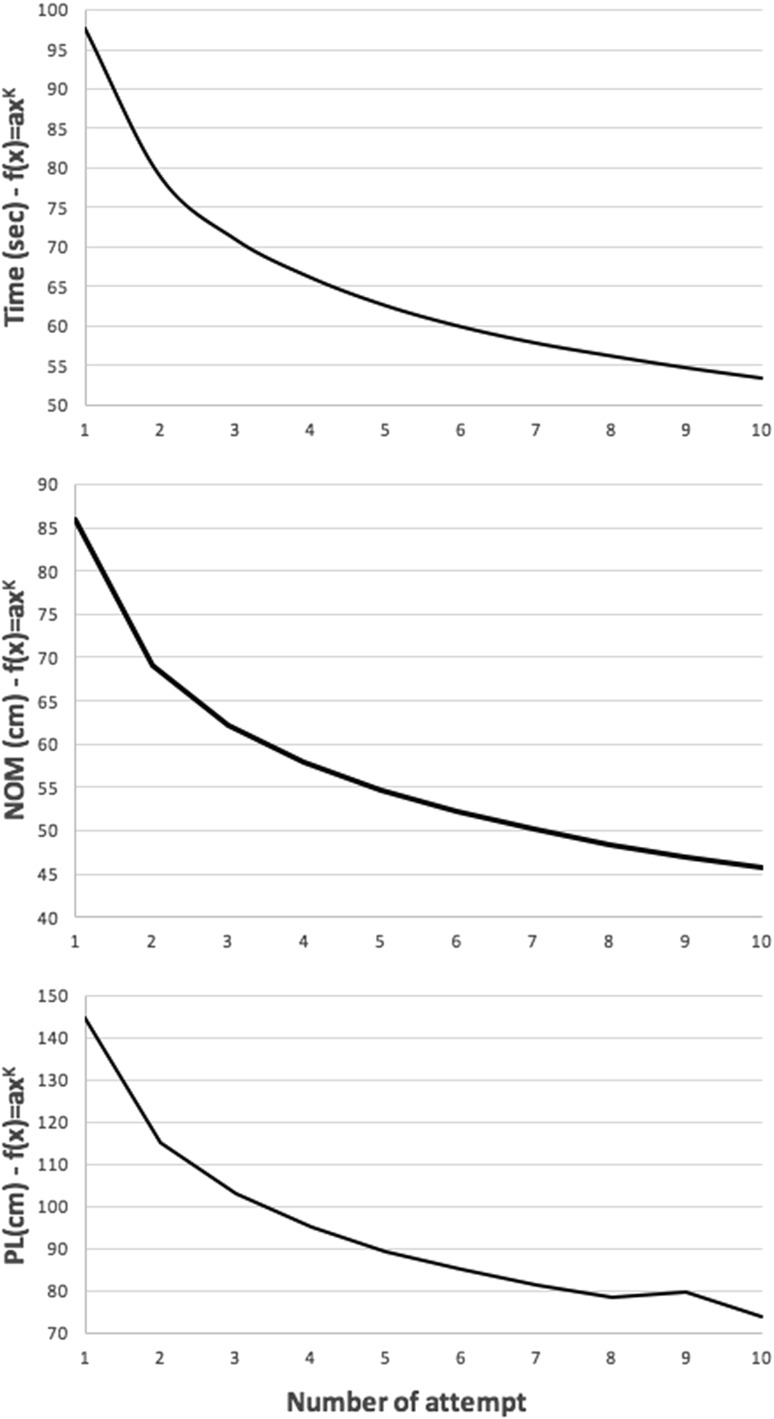
Learning curves for initial 10 LCs

The baseline ability of the two groups was similar (Fig. 3). Proficiency gain curves demonstrated that medical students experienced a learning effect prior to embarking on the comparative part of the study (Fig. 4).

### VRS performance and safety metrics

#### Normal anatomy

**Fig. 5:**
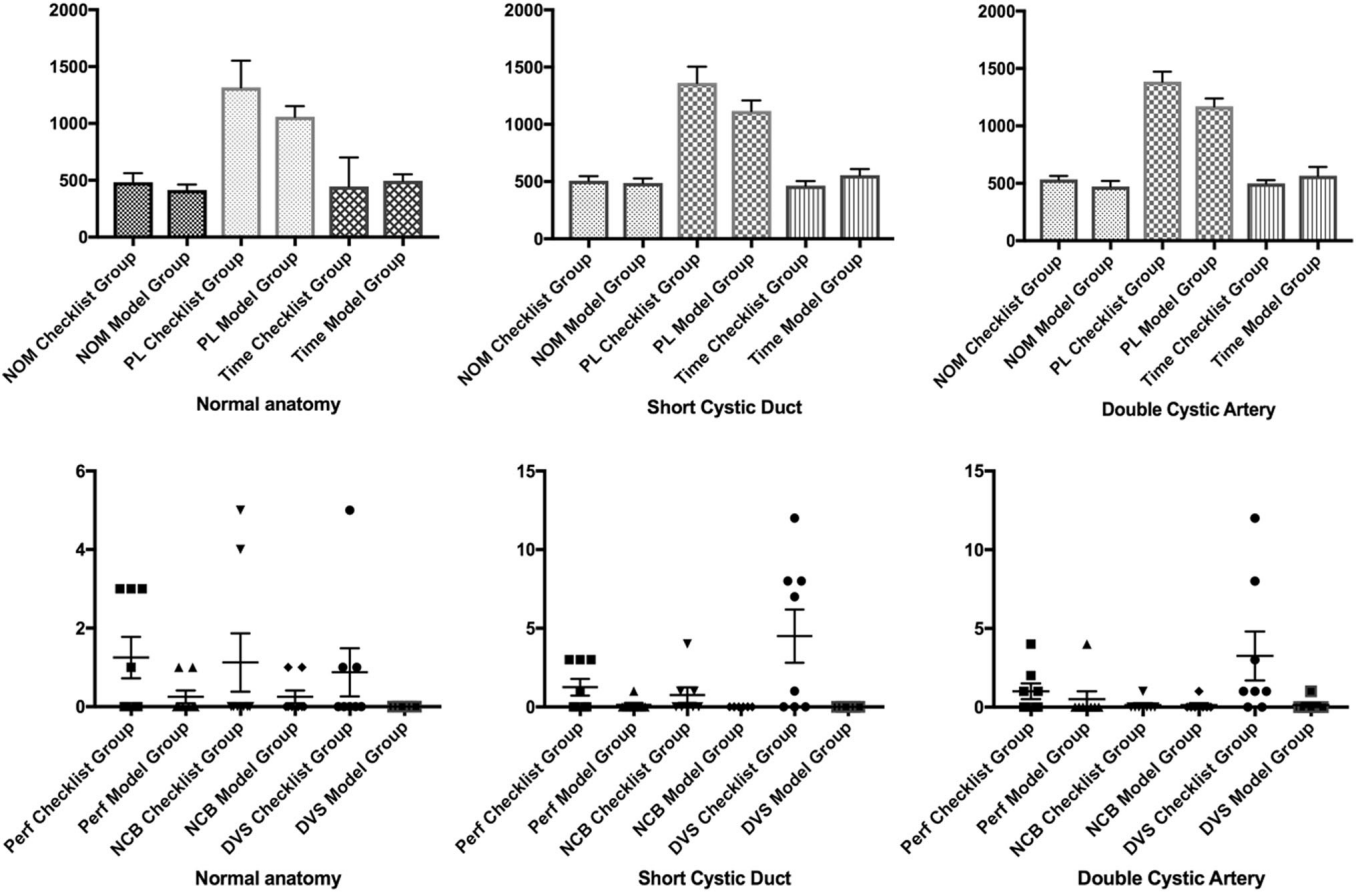
VRS metrics. *NOM* number of movements, *PL* path length. PL is measured in cm and time in secs

There was no statistical difference in performance [checklist vs. model—time (s): 445.5 vs. 496 *p* = 0.64—NOM: 437 vs. 413 *p* = 0.88 – PL [75]: 1317 vs. 1059 *p* = 0.32] or safety metrics [checklist vs. model—per: 0.5 vs. 0 *p* = 0.22—NCB: 0 vs. 0 *p* = 0.71—DVS: 0 vs. 0 *p* = 0.2] between the two groups (Fig. 5).

#### Short cystic artery

There was no statistical difference in all metrics but the number of damage to vital structures that was significantly greater in the Group A [checklist vs. model—time (s): 464.3 vs. 555 *p* = 0.2—NOM: 506 vs. 481 *p* = 0.86—PL [75]: 1363 vs. 1118 *p* = 0.17—per: 0.5 vs. 0 *p* = 0.13—NCB: 0 vs. 0 *p* = 0.2—DVS: 4 vs. 0 *p* = 0.03 *η* ^2^ = 0.34] (Fig. 5).

#### Double cystic artery

The only parameter that showed a significant difference was the number of damage to vital structures in Group A [checklist vs. model—time (s): 498.4 vs. 565.8 *p* = 0.43—NOM: 541.5 vs. 514.5 *p* = 0.4—PL [75]: 1385 vs. 1171 *p* = 0.07—per: 0.5 vs. 0 *p* = 0.28—NCB: 0 vs. 0 *p* > 1—DVS: 1 vs. 0 *p* = 0.02 *η* ^2^ = 0.22] (Fig. 5).

### CAT score

**Fig. 6:**
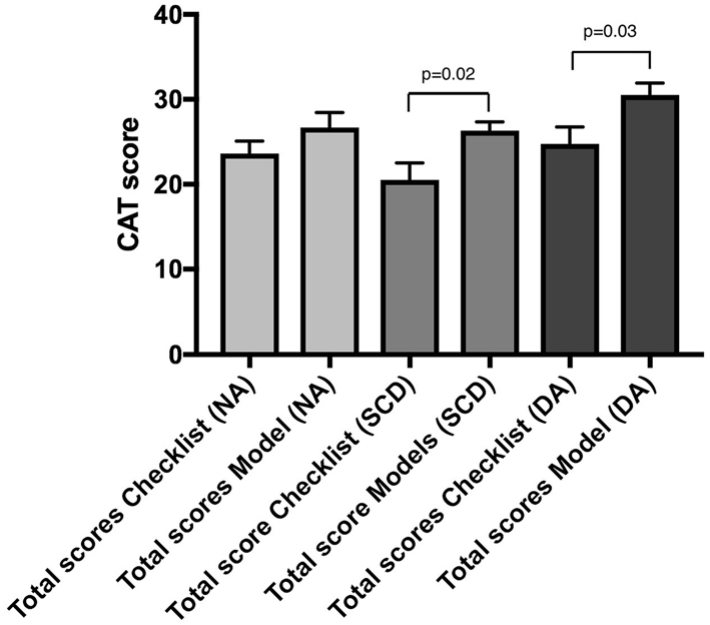
Competency assessment tool scores

The two assessors of the LC videos were in good agreement with each other [ICC: 0.81—95% CI (0.66–0.89)]. According to the CAT scores, Group B performed the SCD and DA LC significantly better than the Group A, but there was no statistically significant difference in the performance of the NA LC [checklist vs. model total CAT score—NA: 23.63 vs. 26.69 *p* = 0.2—SCD: 20.5 vs. 26.31 *p* = 0.02 *η* ^2^ = 0.32—DA: 24.75 vs. 30.5 *p* = 0.03 *η* ^2^ = 0.28] (Fig. 6).

## Discussion

To our knowledge this is the first study testing patient-specific mental rehearsal with the use of 3D interactive visual aids. The results show that both groups performed equally well when given “straight-forward” anatomy that they had encountered before. The group who used patient-specific anatomical models as well as the mental rehearsal checklist performed significantly better (CAT scores) and committed less errors (DVS) in cases with more challenging anatomies (i.e. short cystic duct and double cystic artery). These results support further investigation into the application of patient-specific preparation with the combination of anatomical models and mental rehearsal, within a clinical environment.

The methodology used in this study is aligned to that described in the literature for mental rehearsal [10, 22, 27, 32]. Experts were consulted to create a mental rehearsal checklist and an extensive step-by-step breakdown and teaching and training were provided to the participants prior to the intervention. The performance metrics have been previously validated for demonstrating surgical competency [76]. However, PL and NOM are indicative of economy of movements and any difference in these values may not translate into differences in the safety aspect of the procedure [71]. Similarly, time to complete a procedure is frequently associated with competency [76–79], but not necessarily with quality [71]. This is mirrored in the results of the study, showing completion of the SCD and DA cases in a similar amount of time, whilst Group A had significantly lower CAT score and higher number of damage to vital structures. This justifies the addition of three safety measures (number of perforations, non-cauterised bleeding and damage to vital structures) and the CAT score evaluation as outcome measures. The assessor using CAT score has the opportunity to comment on hazardous use of instruments or detrimental tissue handling, near misses and errors as well as the fluency of the performed operation [71].

This study has some limitations. First, the participants were medical students and not surgeons, which has implications for generalisability. Due to the time commitment needed for the study, it is likely that recruitment of surgical trainees would have resulted in a high drop-out rate, a frequent problem with educational studies [80–82]. Although the authors recognise that medical students are not the target group of the suggested intervention, every possible effort was made to maintain uniform experience and baseline ability of participants (Fig. 3). Second, the study was not conducted in a clinical environment but in a simulation suite. Whilst the VRS used in this study has good validity [83, 84] and skills gained using such simulators are transferable to the operating room [79], there are intrinsic differences between a simulated and a real procedure [71]. This is reflected in the minor modifications needed for the CAT score and mental rehearsal checklist to extract the parts of the procedure not portrayed on the simulator (e.g. insertion of ports or patient positioning). Having established a possible benefit to mental rehearsal combined with patient-specific anatomical models in a simulated environment, the next step is to test the intervention within a clinical randomised controlled trial. The participants in the future trial should be surgical trainees.

## Conclusion

The combination of mental rehearsal and patient-specific anatomical models reduces error occurrence and improves quality of surgery in complex procedures undertaken within a simulated environment.
